# Isolation of Decidual Macrophages and Hofbauer Cells from Term Placenta—Comparison of the Expression of CD163 and CD80

**DOI:** 10.3390/ijms23116113

**Published:** 2022-05-30

**Authors:** Manuel Lasch, Kritika Sudan, Corinna Paul, Christian Schulz, Thomas Kolben, Julia van Dorp, Sibel Eren, Susanne Beyer, Lorenzo Siniscalchi, Sven Mahner, Udo Jeschke, Sarah Meister

**Affiliations:** 1 Department of Otorhinolaryngology, Head and Neck Surgery, University Hospital, Ludwig-Maximillians-Universität München, 81377 Munich, Germany; manuel.lasch@med.uni-muenchen.de; 2 Walter-Brendel-Centre of Experimental Medicine, University Hospital, Ludwig-Maximilians-Universität München, 81377 Munich, Germany; kritika89.sudan@gmail.com (K.S.); corinna.paul@med.uni-muenchen.de (C.P.); christian.schulz@med.uni-muenchen.de (C.S.); julia.van.dorp@hotmail.de (J.v.D.); sibel.eren@med.uni-muenchen.de (S.E.); 3 Biomedical Center, Institute of Cardiovascular Physiology and Pathophysiology, Faculty of Medicine, Ludwig-Maximillians-Universität München, 82152 Munich, Germany; 4 Medizinische Klinik und Poliklinik I, University Hospital, Ludwig-Maximillians-Universität München, 81377 Munich, Germany; lollosini69@gmail.com; 5 Department of Obstetrics and Gynecology, University Hospital, Ludwig-Maximillians-Universität München, 81377 Munich, Germany; thomas.kolben@med.uni-muenchen.de (T.K.); susanne.beyer@med.uni-muenchen.de (S.B.); sven.mahner@med.uni-muenchen.de (S.M.); udo.jeschke@med.uni-muenchen.de (U.J.); 6 Department of Gynecology and Obstetrics, University Hospital Augsburg, 86156 Augsburg, Germany

**Keywords:** placental macrophages, macrophage isolation protocol, Hofbauer cells, decidual macrophages

## Abstract

(1) Background: Placental immune cells are playing a very important role in a successful placentation and the prevention of pregnancy complications. Macrophages dominate in number and relevance in the maternal and the fetal part of the placenta. The evidence on the polarization state of fetal and maternal macrophages involved in both, healthy and pregnancy-associated diseases, is limited. There is no representative isolation method for the direct comparison of maternal and fetal macrophages so far. (2) Material and Methods: For the isolation of decidual macrophages and Hofbauer cells from term placenta, fresh tissue was mechanically dissected and digested with trypsin and collagenase A. Afterwards cell enrichment was increased by a Percoll gradient. CD68 is represented as pan-macrophage marker, the surface markers CD80 and CD163 were further investigated. (3) Results: The established method revealed a high cell yield and purity of the isolated macrophages and enabled the comparison between decidual macrophages and Hofbauer cells. No significant difference was observed in the percentage of single CD163^+^ cells in the distinct macrophage populations, by using FACS and immunofluorescence staining. A slight increase of CD80^+^ cells could be found in the decidual macrophages. Considering the percentage of CD80^+^CD163^−^ and CD80^−^CD163^+^ cells we could not find differences. Interestingly we found an increased number of double positive cells (CD80^+^CD163^+^) in the decidual macrophage population in comparison to Hofbauer cells. (4) Conclusion: In this study we demonstrate that our established isolation method enables the investigation of decidual macrophages and Hofbauer cells in the placenta. It represents a promising method for direct cell comparison, enzyme independently, and unaffected by magnetic beads, to understand the functional subsets of placental macrophages and to identify therapeutic targets of pregnancy associated diseases.

## 1. Introduction

The human placenta is a chimeric and very complex organ, which contains maternal and fetal tissue and immune cells. As a highly specialized organ, it is crucial for a healthy pregnancy for mother and child. One can distinguish between fetal and maternal compartments of the placenta, nevertheless the borders are not very distinct [1].

Fetal stem cells of the trophectoderm develop to primary villi and differentiate to the villous cytotrophoblast and the outer syncytiotrophoblast, forming the placental villi [2]. Further invasive cytotrophoblast cells proliferate and migrate as extravillous trophoblast cells to the maternal endometrium which is transformed to decidual stroma, building together the decidua [3,4]. The syncytiotrophoblast provides the maternal fetal interface and is responsible for the nutrient transport and exchange between the maternal blood which passes through the spiral arteries into the intervillous space [5].

This proximity of mother and semi-allogenic fetus requires a specific and properly adapted immune tolerance towards the fetus to avoid pregnancy complications. Therefore, placental immune cells play a crucial role in preserving a healthy pregnancy. At the maternal–fetal interface, where fetal trophoblast cells invade the maternal decidua and spiral artery remodeling takes place, decidual macrophages and natural killer cells (NK cells) account for the largest and most important immune cell populations together with T-cells [6,7]. These maternal leukocytes, which are functional and phenotypical distinct from their correspondents circulating peripherally, are present in the decidua during whole pregnancy, nevertheless occurrence is changing from first trimester until term: the decidual macrophage and NK cell number is decreasing by time [8,9]. Maternal leucocytes in the decidua are recruited by chemokines which are produced by decidual stroma cells and trophoblasts [10,11,12].

The functions of decidual macrophages during pregnancy are broad: they provide spiral artery remodeling and trophoblast invasion, and might promote angiogenesis and tissue remodeling [13,14,15,16,17,18]. They are proposed to play a distinct role in trophoblast phagocytosis [19,20]. Decidual macrophages have been characterized as mainly anti-inflammatory and regulatory cells of an M2-like phenotype [6,21], producing many factors which are associated with immune modulation: as IL-4, IL-10, and TNFα. Nevertheless, they are not typical M2 macrophages, since they are not typically induced by Th2 cytokines, but by M-CSF and IL-10 [22]. Furthermore there are other studies showing that there are different subpopulations of decidual macrophages which produce either pro- or anti-inflammatory cytokines [23].

There is another subset of maternal placenta macrophages which have been identified by Thomas et al. named as PAMM1 (maternal villous macrophages) [24]. This macrophage population could be subdivided into PAMM1b seeming to have close relation to circulating maternal monocytes and PAMM1a which seem to be very specific for the placental niche and appear to stay in narrow contact with the surface of the placental villi [24].

The most important fetal immune cell subpopulation in the placenta represents the Hofbauer cells, which are fetal tissue resident macrophages occurring exclusively in the villous stroma. However, they were discovered many years ago [25] and are known to be from fetal origin, by the performance of Y-chromosome detection in pregnancies with male fetuses, they remain poorly understood compared to adult tissue resident macrophages. It has been shown that Hofbauer cells play a crucial role in the remodeling of blood vessels by secreting VEGF-A, OPN, MMP-9, and TIMP-1 [26,27,28,29,30]. Further Thomas et al. showed a secretion of factors which are typically associated with inflammation such as IL-8, CCL2, CCL3, and CCL4 [24].

Besides the different subsets of placental macrophages their polarization is crucial for maintaining healthy pregnancy. There are two main polarization profiles: M1-like and M2-like phenotypes which can be distinguished in macrophage populations, leading to the expression of specific surface markers and to the secretion of several cytokines. As their ability of variable polarization states macrophages are known as a high plasticity cell population [31,32]. Regarding this information, the classification in M1- and M2-like polarization profiles only demonstrate extremes in a wide range of possible polarizations. M1-like polarized macrophages are mainly involved in anti-microbial and tumoral activities and release several cytokines such as, TNF-α, IL-1α, IL-1β, IL-6, IL-12, CXCL9, and CXCL10 which exert positive feedback on unpolarized macrophages. Typical surface markers of M1-like polarized macrophages are CD80, CD86, and IL-1R as well as toll-like receptors [33]. CD80 is a costimulatory factor, which is expressed on dendritic cells, T-cells, and macrophages, in the placenta known to be especially expressed on decidual macrophages [34,35].

M2-like polarized macrophages can be subdivided in different subtypes and contribute to processes such as infection prevention, tissue repairing, angiogenesis, and immunomodulation [36]. One M2 typical marker represents CD163, which is induced by anti-inflammatory signals, whereby CD163 also acts as immunomodulator. Further, it is an erythroblast adhesion receptor and a receptor for tumor necrosis factor-like weak inducer of apoptosis. Another function represents the internalization of hemoglobin-haptoglobin complexes. However, its most important role in pregnancy is assumed to play an immunomodulatory role [37,38]. There are various publications having shown partially contradictory findings, in changes of M1- and M2-like polarization in the placenta. There is still a lot of confusion about pro- and anti-inflammatory polarization of fetal and maternal macrophages during healthy pregnancies, and especially during pregnancy-associated diseases such as preeclampsia, intrauterine growth restriction, and gestational diabetes, mainly in term placentas [33,36,39,40]. Especially considering the loss of maternal immunotolerance against the fetus and the combination of inflammatory processes [41,42,43,44], the investigation of pro- and anti-inflammatory markers on placental macrophages, as e.g., CD80 and CD163, is very important to understand the pathophysiology of pregnancy-associated diseases better, and to possibly contribute to the development of future therapy strategies.

For digesting VT, either trypsin or collagenase or a combination of both was used. Tang et al. for example used multiple trypsin digestion steps, followed by a collagenase A step, 2 Percoll gradients and finally a negative immunoselection with magnetic CD10 and EGFR beads to isolate Hofbauer cells [45]. Johnson et al. performed for their Hofbauer cell isolation one digestion step with collagenase IV, then density gradient centrifugation with Histopaque 1077 and positive selection using CD14 beads [46]. One or more digestion steps with trypsin followed by Percoll or Ficoll gradients and magnetic cell selection with CD14 beads was used by Challier et. al. and Mezouar et al. [47,48].

There are few studies concentrating on the isolation of decidual macrophages out of term placenta and we were not able to find a study comparing both subpopulations by using nearly the same isolation process, as we did in this study. Laskewitz et. al. isolated maternal macrophages using Accutase, GentleMACSDissociator, and one Percoll gradient [49]. In most publications Collagenase I was used as enzymatic digestion method. Co et al. used Collagenase I-A for digestion and a Ficoll density gradient followed by magnetic beads [50], similarly to Heikkinen et al. [35]. Vishnyakova et al. used Collagenase I, II for digestion followed by a separation with Lympholyte-H [40].

We aimed to establish a method for the isolation of decidual macrophages and Hofbauer cells from fresh term placenta with the same enzymatic components, but without the usage of magnetic beads to minimize priming of macrophage surface markers in the isolation procedure and to isolate as native as possible macrophages to compare the cells directly after isolation without any changes by substances influencing the macrophages in cell culture. Therefore, we immediately performed FACS analysis after the isolation process and did not put the cells in culture as it has been done by most of the groups concentrating on the characterization of placental macrophages. Loegl et al. e.g., cultured macrophages isolated from diabetes placenta with several culture conditions and coating strategies and FACS analysis was performed 5 days after culture.

We tried to broadly characterize the two macrophage populations by using the pan-macrophage marker CD68, which has been used in several publications before [51,52,53] and to investigate the expression of CD163, being expressed on M2-like polarized macrophages and CD80, which is known as one possible marker for M1-like polarized macrophages [36,40,54,55]. In addition, cytokeratin 7 (CK7) was used as a marker for trophoblasts to verify cell purity of immune cells [45,56].

## 2. Results

### 2.1. High Cell Yield and Cell Purity of the Established Isolation Method

**Figure 1:**
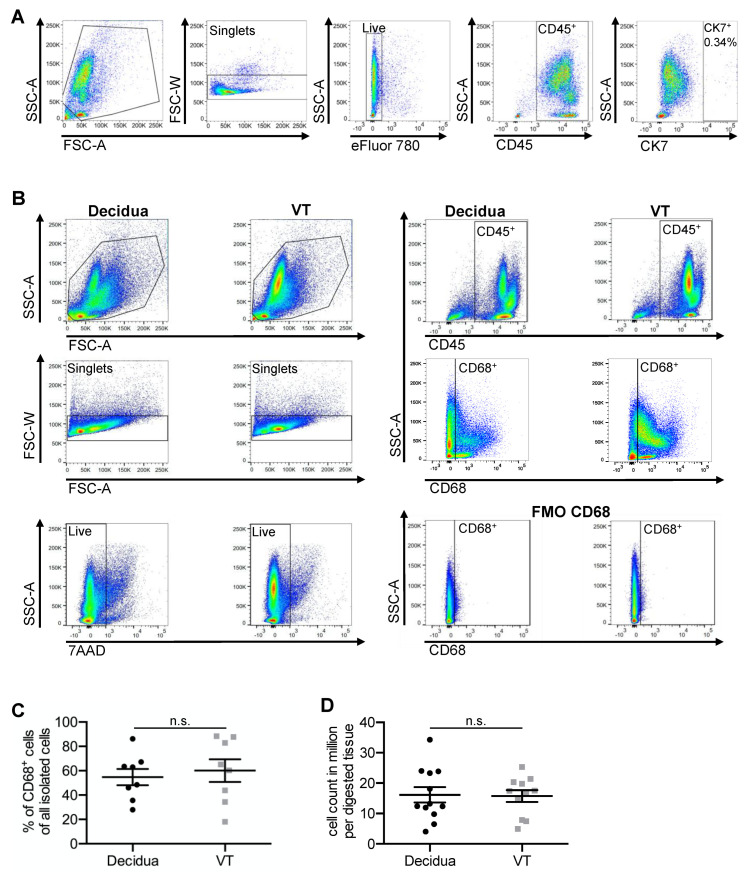
Cell yield and purity—(**A**) FACS images of isolated cells with CK7 as trophoblast marker and the corresponding FMO. (**B**) Representative FACS images of isolated cells from decidua and VT. (**C**) Percentage of CD68^+^ cells in FACS analysis of decidua (35 gr.) and VT (150 gr.). Data are mean ± S.E.M., *n* = 8, n.s. *p* > 0.05 (decidua vs. VT) by Mann–Whitney-U-Test. (**D**) Dotplots of cell counts in million per digested tissue (35 gr. decidua and 150 gr. VT) of the immune cell layer. Data are mean ± S.E.M., *n* ≥ 11, n.s. *p* > 0.05 (decidua vs. VT) by Mann–Whitney-U-Test.

To check the purity of isolated immune cells including macrophages and whether there was any trophoblast contamination, CK7 staining of the immune cell layer was performed. As shown in Figure 1A, there were almost no trophoblasts (0.34%). After the isolation process, cell counting was performed by using viability dye. The number of intact isolated immune cells was comparable between the decidual and the villous tissue (VT) (*p* = 0.913). Per digested tissue, 16.1 × 10^6^ (±2.5 × 10^6^) immune cells could be isolated from the decidual tissue and 15.7 × 10^6^ (±1.9 × 10^6^) immune cells from the VT (Figure 1D). The isolated cell suspension showed approximately the same percentage of CD68^+^ cells (*p* = 0.702). The percentage of CD68^+^ cells from all isolated cells accounted for 60.1% (±9.3%) from VT and 54.7% (±6.7%) from decidua (Figure 1B,C).

To correlate our FACS data with another analysis method, we performed an immunohistochemical staining for CD68, where data retrieved from the FACS analysis correlated with immunohistochemical staining. The mean cell count of CD68^+^ cells per visual field differed significantly (*p* = 0.011 *) between decidua and VT, as the number of CD68^+^ cells in the decidua (9.7 (±1.7)) was higher compared to the VT (3.6 (±0.8)) (Supplementary Figure S4).

### 2.2. Correlation of CD68^+^ Cells and Clinical Features

In summary, we achieved to obtain similar results concerning the total cell yield and the percentage of macrophages in the isolated immune cell populations comparing Hofbauer cells and decidual macrophages. However, the variability of the percentage of CD68^+^ cells in the subgroups is high, which needs to be further elucidated. Only placentas from healthy women were used to establish the isolation procedure, however it is necessary to evaluate whether these differences refer to possible alterations in the placentas which were used for our experiments. To explore the reasons in depth we analyzed clinical differences between the patients, as the weight of the baby, gender of the baby, and pregnancy week to find possible correlations. There could not be found a significant relevance but a quite high correlation of the gestational age which might be a possible explanation (Supplementary Table S1). The spearman correlation analysis of isolated CD68^+^ cells and several patient characteristics (gestational age, fetal sex, fetal birth weight, maternal age, and delivery mode) revealed neither in the decidua nor in the VT a significant correlation (Supplementary Figure S2 and Tables S1 and S2).

### 2.3. Differences between Decidual and Hofbauer Macrophages in Expressing Pro- and Anti-Inflammatory Surface Markers

**Figure 2:**
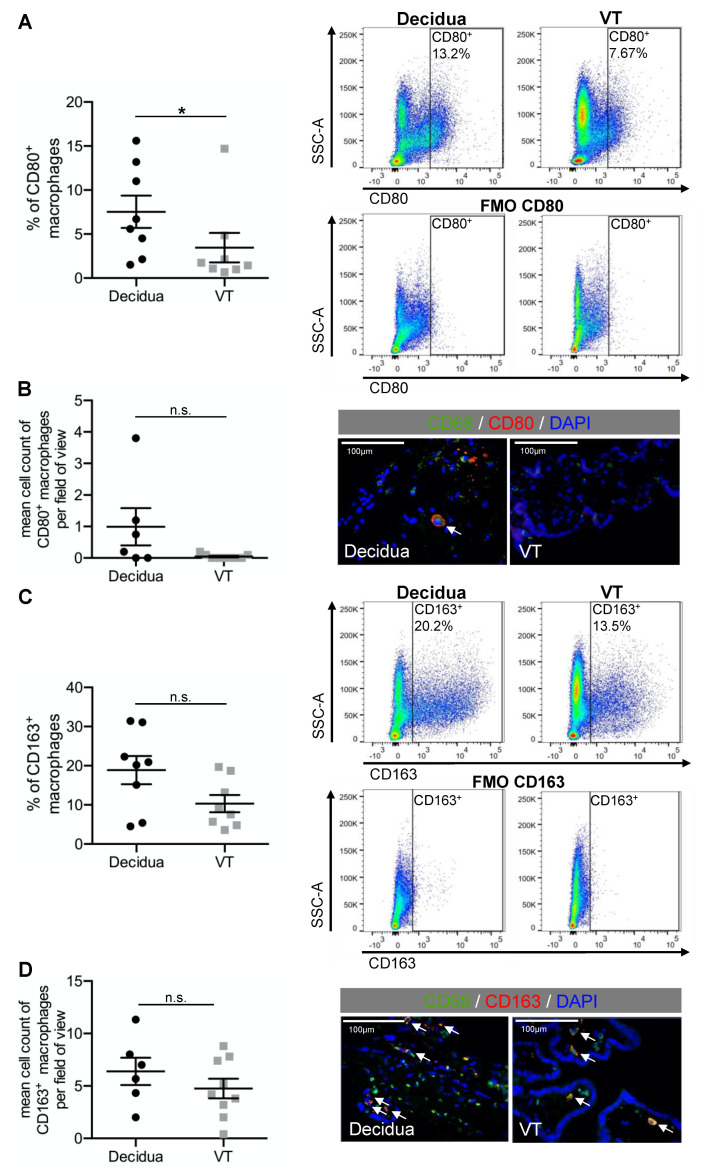
Expression of pro- and anti-inflammatory surface markers in decidual macrophages and Hofbauer cells (VT). (**A**) Dotplots of the left graph show the percentage of CD80^+^ macrophages with representative FACS images on the right side. (**B**) Dotplots of the left graph show the mean cell count of CD80^+^ macrophages per visual field with representative IF images on the right side. (**C**) Dotplots show the percentage of CD163^+^ macrophages with representative FACS images on the right side. (**D**) Dotplots show the mean cell count of CD163^+^ macrophages per field of view with representative IF pictures on the right side. Data are mean S.E.M., *n* = 8 (FACS) *n* ≥ 6 (IF), * *p* < 0.05, n.s. *p* > 0.05 (decidua vs. VT) by Mann–Whitney-U-Test.

**Figure 3:**
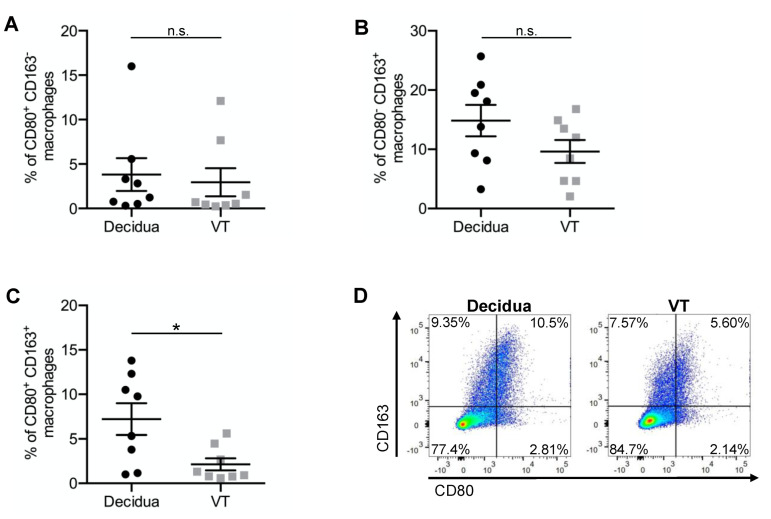
Analysis of pro- and anti-inflammatory surface marker expression in decidual macrophages and Hofbauer cells (VT). Dotplots show the percentage of (**A**) CD80^+^CD163^−^ macrophages, (**B**) CD80^−^CD163^+^ macrophages and (**C**) CD80^+^CD163^+^ macrophages. (**D**) representative FACS images of decidual macrophages and Hofbauer cells (VT). Data are mean ± S.E.M., *n* = 8, * *p* < 0.05, n.s. *p* > 0.05 (decidua vs. VT) by Mann–Whitney-U-Test.

Both, the percentage of CD80^+^ (*p* = 0.041 *) and of CD80^+^CD163^−^ (*p* = 0.433) macrophages was increased in the decidua compared to the VT. 7.5% (±1.8%), of the decidual macrophages were CD80^+^ and 3.8% (±1.9%) were CD80^+^CD163^−^. In the VT 3.5% (±1.7%) of the macrophages were CD80^+^ and 2.9% (±1.6%) were CD80^+^CD163^−^ (Figure 2A and Figure 3A). The immunofluorescence staining showed an increase of CD80^+^ macrophages in the decidua (0.99 (±0.6)) compared to the VT (0.04 (±0.0)) (*p* = 0.066) (Figure 2B) as well.

In addition, the percentage of CD163^+^ (*p* = 0.083) and of CD80^−^CD163^+^ (*p* = 0.159) macrophages was slightly but not significantly increased in the decidua compared to the VT. 18.9% (±3.6%) of the macrophages in the decidua were CD163^+^ and 14.8% (±2.7%) were CD80^−^CD163^+^. In the VT, 10.3% (±2.2%) of the macrophages were CD163^+^ and 9.6% (±1.9%) were CD80^−^CD163^+^ (Figure 2C and Figure 3B). Additionally, the immunofluorescence analysis revealed no significant difference in the number of CD163^+^ macrophages, however there was a slight increase in the number of CD163^+^ macrophages in the decidua (6.4 (±1.3)) than in the VT (4.8 (±0.9)) (*p* = 0.343) (Figure 2D).

Furthermore, the FACS staining showed that CD80^+^CD163^+^ macrophages were increased significantly (*p* = 0.028 *) in the decidua (7.2% (±1.8%)) compared to the VT (2.1 (±0.7%)) (Figure 3C,D).

## 3. Discussion

There are several different protocols for the isolation of maternal and fetal macrophages from fresh term placenta. In previous studies, placental macrophages were isolated using various enzymatic digestion steps, density gradients, and immunoselection, mostly by magnetic bead selection and mainly from first trimester placenta.

We established an easily reproduceable and direct comparable isolation method to isolate decidual macrophages and Hofbauer cells out of term placenta, independently from magnetic beads, using the same enzymatic components and were able to characterize differences in the expression of CD163 and CD80 by FACS analysis.

Current evidences, have either utilized sequential enzymatic digestion or single enzymatic digestion of trypsin and collagenase for the isolation of placental macrophages. This step has not been comparably applied for Hofbauer cells and decidual macrophages in any study before.

Our aim was to establish a standardized method to isolate Hofbauer cells as well as decidual macrophages and to minimize enzyme dependent changes of either surface markers or epigenetics. Therefore, we used the same enzymes for Hofbauer cells and decidual macrophages to achieve optimal comparable results.

In nearly every protocol for the isolation of placental macrophages, which can be found in the literature, magnetic beads were used to achieve a higher macrophage purity and to avoid trophoblast contamination. Therefore, either negative selection by EGFR beads or positive selection by e.g., CD14 beads was performed [47,48,54]. There, good cell purity results were achieved. However, there are several studies showing a not neglecting influence of magnetic beads on the phenotype and function of isolated immune cells [57,58,59]. Therefore, we dispensed to use magnetic beads either in positive or negative selection. A certain pipetting technique which was developed and is explained in our trouble shoot selection was used to avoid trophoblast contamination in our immune cell suspension. By performing FACS analysis we could show that our isolated immune cell populations were not contaminated by trophoblast cells, just by performing enzymatic digestion followed by a Percoll gradient. By dispensing magnetic bead purification, we avoided further cell activation without accepting a loss of purity.

To separate both macrophage populations from each other, mechanical dissection of the tissue is the most important and crucial step. Decidual macrophages are located at the maternal–fetal interface, where trophoblast invasion and spiral artery remodeling takes place [60]. Their number is variant depending on the week of pregnancy and is reduced in term placenta compared to first trimester placenta. Hofbauer cells are located in the chorionic villi nearby the villous vessels, their contribution varies between term and first trimester placenta [36,61]. Thomas et al. was able to differentiate distinct macrophage populations in first trimester placenta by using CD45 and CD14 as main markers to identify placental macrophages. The group was able to differentiate between three different macrophage subpopulations: Hofbauer cells, PAMM1 (maternal villous macrophages) and PAMM2 (maternal decidual macrophages). The insight, on which their analysis is based, is the expression of HLA-DR having been identified to be specific for maternal cells, by using specific anti-HLA antibodies (instead of sex chromatin staining) in first trimester placenta. These were termed as PAMMs, consisting of two major populations HLA-DR^hi^/^lo^FOLR2^−^ (PAMM1) and HLA-DR^hi^FOLR2^hi^ cells (PAMM2). They compared PAMM2 and maternal blood cells and found a phenotypical concordance. However, there are data indicating that decidual macrophages may originate not only form hematopoietic stem cells-derived macrophages but from embryonic macrophages as well [62]. Heikkinen et al. found higher levels of HLA-DR on decidual macrophages compared to macrophages isolated from blood. Nevertheless, there are no specific markers in term placenta so far to distinguish between Hofbauer cells and decidual macrophages apart from placental-tissue localization, since there is various data indicating that the expression of macrophage markers is varying during pregnancy [47,55,63]. Thomas et al. postulate HLA-DR negativity for Hofbauer cells in preterm placenta whereby HLA-DR was found on Hofbauer cells of term placenta in other studies [64,65]. Therefore, the establishment of a standardized isolation process to make out differences between the macrophage subpopulation in term placenta is very important and should be an aim to be set up.

In our study, by using nearly the same protocol for decidual macrophages and Hofbauer cell isolation—which has not been performed in such manner by other groups—the comparison of the cell yield in the different placental tissue parts was possible. The number of isolated cells differed not significantly between the two groups. When considering the amount of tissue we used for the digestion, there was a higher amount of decidual macrophages per gram digested tissue compared to Hofbauer cells. These findings could be confirmed by immune fluorescence staining. Al-khafaji et al. determined the number of placental macrophages of control and preeclamptic women, there they found a slightly higher amount of decidual CD68^+^ cells per visual field, but they did no statistical comparison [66]. Another group digested whole term placenta tissue and found that 30% of all macrophages were of maternal origin whereby 70% were of fetal origin by performing fluorescence in situ hybridization. This is in accordance with our data, considering that the proportion of decidual tissue out of the whole placenta is lower than 30% [67].

To investigate possible differences in the expression of surface markers, being predominantly expressed in either M1-like or M2-like polarized macrophages FACS analysis was performed. CD163 and CD80 were chosen, as they are either well established in FACS and immunofluorescence staining [68,69,70], which was used as method to verify the applicability of the established isolation protocol. CD163 represents a marker which is associated with M2-like polarization (M2a and M2c) [63,71]. Comparing both populations, we could not find a significant difference of CD163^+^. There were no comparable results in the literature, however CD163 is expressed either in Hofbauer cells and decidual macrophages, nevertheless CD163 is better known for typical M2-like polarized Hofbauer cells [72,73].

CD80 is known as one marker being associated with M1-like polarization. Further CD163 as well as CD80 are known to be expressed by decidual macrophages [6].

The percentage of CD80^+^ cells showed a slight increase in decidual macrophages compared to Hofbauer cells, however the MFI differed, not significantly, between the distinct macrophage populations. In accordance with other studies, we found low numbers of CD80^+^ cells in the Hofbauer cell population and the decidual cells [35,74]. Further, in our FACS analysis no significant difference could be found in the expression of CD163 between decidual and Hofbauer macrophages. These results could be reproduced by immunofluorescence staining.

Considering the percentage of CD80^+^CD163^−^ and CD80^−^CD163^+^ cells we could not find differences between decidual macrophages and Hofbauer cells. Interestingly we found differences in double positive cells (CD80^+^CD163^+^) in the analyzed macrophage populations. Pointing to the theory that decidual placental macrophages are able to express pro-inflammatory and anti-inflammatory markers. In accordance with Houser et al. who found a decidual cell population which does not fit conventional M1/M2 categorization [23]. The percentage of double positive cells was higher in decidual macrophages compared to Hofbauer cells, however they used preterm placenta. During the peri-implantation period decidual macrophages are related to M1-like polarized macrophages, afterwards when extravillous trophoblast cells invade the uterine stroma, they transition to a profile of mixed M1- and M2-like polarized macrophages [75]. Until an adequate placental-fetal blood supply is established decidual macrophages remain M1- and M2-like mixed macrophages. Afterwards they shift towards a predominantly M2 phenotype to maintain immune tolerance against the fetus [76], as they are the major source of IL-10 in the decidua, confirming their role in immunosuppression [77]. However, in several pregnancy diseases, as gestational diabetes [33,68,78], preeclampsia [79,80,81], and fetal growth restriction [82], literature points to a change of macrophage polarization in line with M1-like polarized macrophages. The increased level of CD80^+^CD163^+^ cells in our study might point to a high plasticity of decidual macrophages being able to shift towards either M1- or M2-like polarization. In accordance with Schonkeren et al. who showed that decidual macrophages at term encode for markers of classical macrophage activation and alternative macrophage activation [39].

However, we achieved to establish an isolation method enabling the comparison of decidual macrophages and Hofbauer cells from term placenta in a, as less as possible activated and differently influenced form, there are limitations left to be discussed. Even though purification of our cell population was verified against trophoblasts, the purity relative to other leucocytes, as performed by other groups [83], would be interesting to be analyzed. Furthermore, analysis of CD163 and CD80 is not sufficient to make an exact statement of M1- or M2-like polarization, which was not the aim of this study so far, but was performed to demonstrate that by using our method macrophage polarization analysis might be one possible application purpose.

In summary, we were able to establish an isolating method for decidual macrophages and Hofbauer cells which allows a direct, enzyme independent comparison of both macrophage populations. We did not use magnetic beads and did not fix or culture the cells before analysis, which preserves the native status of the cells from the placenta. This allows to generate a better understanding of placental macrophages in pregnancy associated diseases such as preeclampsia and gestational diabetes. We elaborated variations in the expression of the surface markers CD163 and CD80, of decidual macrophages and Hofbauer cells in healthy pregnancy, where only few significant results could be found, according to a slightly increased expression of CD80 and CD80CD163 double positive cells in the decidual macrophage population pointing to a high polarization plasticity of decidual macrophages.

## 4. Materials and Methods

### 4.1. Placental Tissue

Placentas from at term deliveries (ceasarean section or vaginal deliveries), of healthy, uncomplicated pregnancies were used. Exclusion factors were obesity, diabetes, hypertension, preeclampsia or COVID-19 disease in the medical history. For patient characteristics refer Table S5 in Supplementary Materials. The placentas were stored at 4 °C and processed as soon as possible.

The study was approved by the ethics committee of the LMU Munich and all the mothers gave written informed consent.

### 4.2. Materials—Macrophage Isolation

0.9% NaCl (Carl Roth, Karlsruhe, Germany) was diluted in ultra-pure water to obtain an isotonic liquid for washing. HBSS-HEPES-buffer was prepared using 25 mM HEPES (Sigma-Aldrich, St. Louis, MO, USA) in 1× HBSS (Life Technologies, Carlsbad, CA, USA) at pH 7.4 to be used as a digestion buffer. DMEM (Sigma-Aldrich, St. Louis, MO, USA) was supplemented with 10% FBS (Sigma-Aldrich, St. Louis, MO, USA) and 1% P/S/A (penicillin/streptomycin/amphotericin) (Life Technologies, Carlsbad, CA, USA) also as digestion solution. Trypsin (Sigma-Aldrich, St. Louis, MO, USA) was diluted in 1 mM HCl at pH 3. DNAse I (Roche, Basel, Switzerland) was prepared in ultra-pure water. Collagenase A (Roche, Basel, Switzerland) was diluted in 1× HBSS. Further, 90% Percoll (Cytiva, Marlborough, MA, USA) was prepared in HBSS-HEPES buffer to create the density gradient.

### 4.3. Materials—FACS Staining

FACS staining buffer, 0.5% albumin (Carl Roth, Karlsruhe, Germany) and 2 mU EDTA (Sigma Aldrich, St. Louis, MO, USA) were dissolved in D-PBS (Life Technologies, Carlsbad, CA, USA). Permeabilization-buffer was comprised of 0.1% saponin (Carl Roth, Karlsruhe, Germany) and 5% FBS (Sigma-Aldrich, St. Louis, MO, USA) in D-PBS. Human serum (PAN biotech, Aidenbach, Germany) was heat inactivated in diluted to 10% in D-PBS. 1% PFA (Thermo Scientific, Waltham, MA, USA) was diluted in D-PBS.

### 4.4. Materials—Immunofluorescence and Immunohistochemistry Staining

For the sodium-citrate-buffer, 18 mL of solution A, 82 mL of solution B, and 900 mL of distilled water were mixed. Solution A was prepared from 21.021 gr. 0.1 citric acid (Merck, Darmstadt, Germany) and 1 L distilled water. For solution B, 29.41 gr. 0.1 M sodium citrate (Merck, Darmstadt, Germany) was dissolved in 1 L distilled water.

### 4.5. Protocol—Macrophage Isolation

#### 4.5.1. Dissection of the Decidua Basalis

**Figure 4:**
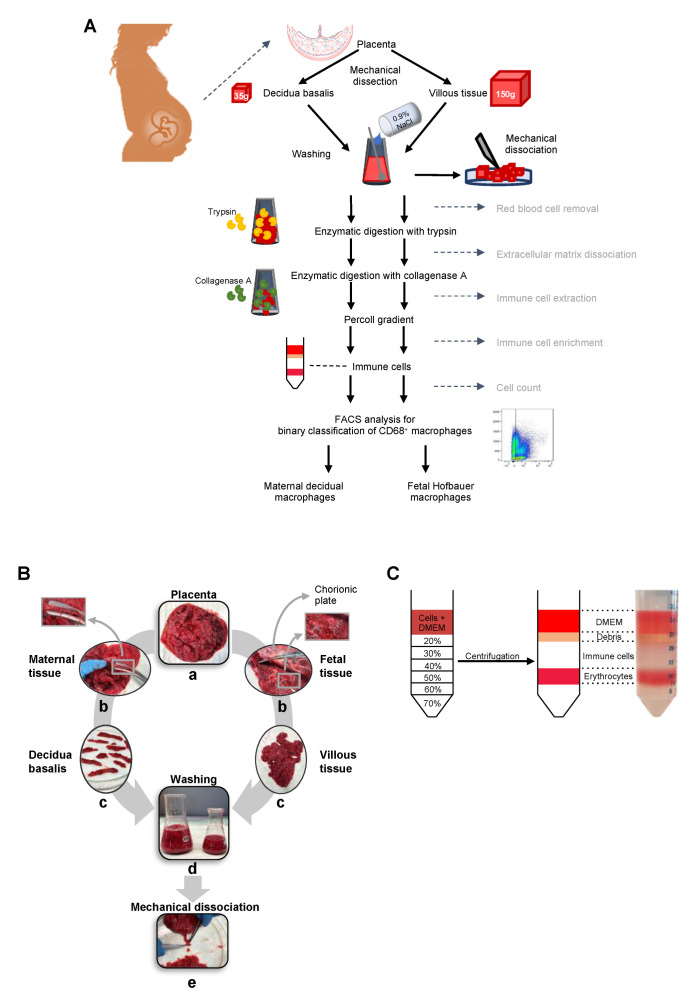
(**A**) Graphical abstract of the isolation protocol. (**B**) Mechanical dissection of the placenta: a, Whole placenta, b, dissection of decidua basalis and VT with enlarged details, c, dissected decidual and VT, d, washing, and e, mechanical dissociation. (**C**) Percoll-layer before centrifugation and the separated layers after centrifugation.

- The superficial residual blood was gently removed with fleece cleaning cloth (WypAlls, Kimberly Clark, Dallas, TX, USA). Then the placenta was placed, with the Decidua basalis facing up, on a cutting board (Figure 4B(a)).
- Scissors and forceps were used to carefully dissect the decidua basalis from the VT (Figure 4B(b)).
- If necessary, the residual villous was removed from the decidua pieces with a very fine scissors.
- The decidua basalis was placed in a glass petri dish (Figure 4B(c)).
- The tissue was weighed and some ml of HBSS was put on it, to prevent it from drying.

#### 4.5.2. Dissection of the VT from the Decidua Parietalis and Chorioamniotic Membranes

- After dissecting the decidua basalis from the placenta, the organ was placed with the chorioamniotic membranes facing upwards. Then the single cotyledons were cut 4 mm above the chorioamniotic membranes (Figure 4B(b,c)).
- The cotyledons were put on a glass petri dish and the tissue was weighed.
- From here on the tissue was treated separately.

#### 4.5.3. Washing and Mechanical Disaggregation

- 150 gr. VT and whole decidua basalis (ca. 35 gr.) were used to go on with digestion.
- The tissue was scratched with a scalpel to create a rough, mesh-like surface.
- Afterwards the tissue was put in Erlenmeyer flasks and washed several times with fresh cold 0.9% NaCl by stirring, until the solution gets nearly clear (Figure 4B(d)).
- The last wash step was performed with HBSS (Life Technologies, Carlsbad, CA, USA).
- Tissue was placed back on the glass petri dish and cut as mince as possible with a sharp scalpel (Figure 4B(e)).

#### 4.5.4. Enzymatic Digestion

- For enzymatic digestion the tissue was transferred into glass bottles (250 mL for decidua, 500 mL for VT) with representative digestion solution (50 mL for decidua, 50 mL for VT).
- The different digestion steps were followed for both tissue parts as described in Supplementary Tables S3 and S4. This was performed following Tang, Tadesse et al. [34].
- For the whole decidua (ca. 35 gr.) and for 150 gr. VT 50 mL of digestion solution were used.
- After each trypsin-digestion step the digested tissue was put onto a fleece cleaning cloth in a funnel and washing was performed with HBSS-HEPES using the fleece cleaning cloth as a filter. The tissue was put back into the bottle and the next digestion solution was added.

#### 4.5.5. Percoll Gradient

- After the last digestion step the tissue was transferred to 50 mL tubes and the tubes were pulse centrifuged (2100× *g* for 1 min). The supernatant was taken off and filtered through a 100 µm cell strainer. Digestion was stopped with 1/10th of cold FACS-staining-buffer (add 10 mL ice cold FACS-staining-buffer to 100 mL digested solution).
- The cell pellet was centrifuged down and washed with DMEM once. The cells from VT were resuspended in 32 mL and the cells from decidua in 16 mL DMEM.
- Percoll-Layer with 20%, 30%, 40%, 50%, 60%, and 70% Percoll-HBSS-HEPES-solution from 90% Percoll were prepared, by layering 4 mL of each Percoll-solution on the bottom of a 50 mL tube with a 20 G needle on a 5 mL syringe in ascending order (Figure 4C).
- 8 mL of the cell suspension were put on one Percoll-layer and centrifuged 10 min at RT at 2100× *g* without brake.
- With this density gradient macrophages were separated clearly between cellular debris and RBCs as shown in Figure 4C. The first yellowish layer with cell debris was removed with a plastic Pasture pipette and discarded. Afterwards the transparent layer underneath, which was containing the macrophages, was taken off for further use.
- These cells were washed with DMEM, resuspended in FACS-staining buffer and counted in Neubauer counting chamber by usage of trypan blue.

### 4.6. Flow Cytometry

Fc receptors were blocked with 10% human serum for 20 min at RT. Cell surface staining was performed in FACS-staining-buffer (D-PBS, 0.5% albumin, 2 mU EDTA) with CD45-FITC, CD163-APC, and CD80-BV421 for 20 min at 4 °C. Following surface staining, intracellular staining was performed with CD68-APC-Cy7 (and, also, with CD163-APC for CD163 total-staining) in permeabilization-buffer (D-PBS, 0.1% saponin, 5% FBS) for 7 min at RT. Cells were washed with FACS-staining-buffer after each staining step. At the end, the cells were resuspended in FACS-staining-buffer and stored on ice. Cells were acquired in FACS-staining-buffer using 7AAD viability dye. Flow cytometry was performed on BD FACSCanto II and analyzed with FlowJo version 10. During cell gating, the cells were initially selected by size using FCS and SSC. The gating strategy included exclusion of doublets and dead cells. The signal of the certain markers was plotted against the SSC area. For the analysis FMOs and unstained samples were used as negative control for each antibody.

### 4.7. Immunofluorescence Staining

The immunofluorescence staining was carried out on paraffin placenta sections, which were dewaxed for 20 min in Roticlear (Roth, Karlsruhe, Germany). They were then dehydrated in 100%, 70%, and 50% ethanol (CLN GmbH, Freising, Germany) to distilled water. To unmask the proteins, the slides were treated with sodium-citrate-buffer in a pressure cooker. The blocking was carried out for 15 min with the help of UltraVision-Protein-Block (Thermo Fisher Scientific, Waltham, MA, USA). The primary antibodies CD68 (Sigma Aldrich, Saint Louis, MO, USA), CD80 (Sigma Aldrich, Saint Louis, MO, USA), and CD163 (Sigma Aldrich, Saint Louis, MO, USA) were diluted in dilution medium (DAKO, Aligent Technologies, Santa Clara, CA, USA), applied to the sections and incubated in the fridge at 4 °C for 16 h. Then the two secondary antibodies Goat-Anti-Rabbit Cy3 (Dianova, Hamburg, Germany) and Goat-Anti-Rabbit Alexa Fluor 488 (Dianova, Hamburg, Germany) were also mixed together in dilution medium and incubated for 30 min at RT in the dark. Finally, the slides were dried and covered with mounting medium for fluorescence with DAPI (Vector Lab, Burlingame, CA, USA). To ensure that the staining did not stain unspecifically, a negative control with an IgG antibody was also carried out.

### 4.8. Immunohistochemistry Staining

For the immunohistochemistry staining the paraffin placenta sections were dewaxed at first for 20 min in Roticlear and washed in 100% ethanol. To block endogenous peroxidases, sections were incubated for 20 min in 6% H_2_O_2_ (Roth, Karlsruhe, Germany). Then they were dehydrated in 100%, 70%, and 50% ethanol to destilled water. For protein unmasking, the slides were cooked with sodium-citrate-buffer. The slides were blocked at first with ZytoChem-Plus HRP Polymer Kit (Mouse/Rabbit) Reagent 1 (Zytomed Systems GmbH, Berlin, Germany). The primary antibody CD68 (Sigma Aldrich, St. Louis, MO, USA) was diluted in PBS, put on the sections and incubated in the fridge at 4 °C for 16 h. Then they were treated with ZytoChem-Plus HRP Polymer Kit Reagent 2 and with ZytoChem-Plus HRP Polymer Kit Reagent 3. For the enzymatic reaction with the peroxidase contained in the HRP Polymer, the slides were incubated with DAB (Aligent Technologies, Santa Clara, CA, USA) for 2 min and the stopped with aqua dest. The nuclei were stained with Mayer’s acidic hemalaun (Waldeck, Münster, Germany) for 2 min and blued in tap water. For final dehydration, an ascending alcohol series was followed with 70%, 90% and 100% ethanol to Roticlear and subsequent coverslipping of the slide with Roti-Mount (Roth, Karlsruhe, Germany) coverslip medium. A negative control was also performed with an IgG antibody.

### 4.9. Immunohistochemistry Analysis

For evaluation, the number of positive cells of 5 visual fields each from the villous part and 3 visual fields each from the decidual part were counted two different times each under the light microscope (Zeiss, Oberkochen, Germany) with 40× magnification. The mean value of each slide and its maternal or fetal part of each time was calculated, and the mean value of it was taken at the end. The fields of view were always randomly selected for this purpose. Pictures for the s were taken with the help of Flexcam C1 (Leica Microsystems, Wetzlar, Germany) in 40× magnification.

### 4.10. Statistical Analysis

SPSS version 26 and Graph Pad Prism version 9 were used for statistical analysis and the presentation of graphs. Dotplots contain mean ± standard error of the mean (S.E.M.) and the text lists mean ± S.E.M. The non-parametric Mann–Whitney-U-test was performed to compare decidual and Hofbauer macrophages. Non-parametric Spearman correlation analysis was used to check for correlation. The two-sided significance was always calculated and *p*-values below 0.05 were considered to be statistically significant.

## 5. Conclusions

In our study we were able to establish a macrophage isolation method from term placental tissue without the usage of magnetic beads with merely density gradient purification, which allows to compare decidual macrophages and Hofbauer cells characteristics. By the analysis of the expression of the surface markers CD80 and CD163, we showed that our method allows the comparison of the distinct macrophage populations. This achievement enables the investigation of the role of placental macrophages not only in healthy but also in disordered pregnancies, enriching the investigation of pathophysiological processes associated to placental macrophages.

## Data Availability

The datasets generated during the current study are available from the corresponding author on reasonable request.

## Supplementary Materials

The following supporting information can be downloaded at: https://www.mdpi.com/article/10.3390/ijms23116113/s1.

## Abbreviations

CK7	cytokeratin 7
CCL	CC-chemokine ligand
CXCL	chemokin C-X-C-motif ligand
DAB	chromogenic 3,3′-diaminoenzidine
DAPI	4′,6-Diamino-2-phenylindile
DMEM	Dulbecco’s modified eagle’s medium
EDTA	ethylenediaminetetraacetic acid
EGFR	epidermal growth factor
FACS	fluorescence-activated cell sorting
FBS	fetal bovine serum
FMO	fluorescence minus one
FOLR2	folate receptor beta
FSC	forward scatter
HBSS	Hank’s balanced salt solution
HCl	hydrochloric acid
HLA	human leukocyte antigen
HLA-DR	MHC class II cell surface receptor
IL	interleukin
M-CSF	macrophage colony-stimulating factor
MMP	matrix metallopeptidase
NaCl	sodium chloride
NK cells	neutral killer cells
OPN	osteopontin
PAMM	placenta-associated maternal macrophages
PBS	phosphate buffered saline
PFA	perfluroalkoxy alkanes
P/S/A	penicillin/streptomycin/amphotericin
RBCs	red blood cells
RT	room temperature (21 °C)
SSC	side scatter
TNFα	tumor necrosis factor alpha
VEGF	vascular endothelial growth factor
VT	villous tissue
