# Supplementary material for: Isolation of Decidual Macrophages and Hofbauer Cells from Term Placenta—Comparison of the Expression of CD163 and CD80
Source: Int J Mol Sci. 2022 May 30;23(11):6113. doi: 10.3390/ijms23116113 (PMC9181726; doi:10.3390/ijms23116113)
Supplement: Supplementary file 1 [file ijms-23-06113-s001.zip › ijms-1722023-supplementary.pdf]

Supplement

Decidua

	Gestational age	Sex	Birth weight	Maternal age	Birthmode
**Spearman r**	-0.452	0.109	-0.381	0.095	-0.218
**p value**	0.268	0.886	0.360	0.840	0.686

Supplementary Table S1: Spearman correlation of the percentage of $CD68^+$ cells of all isolated decidual cells and different patient characteristics; * significant $p < 0.05$.

Hofbauer

	Gestational age	Sex	Birth weight	Maternal age	Birthmode
**Spearman r**	0.310	0.327	0.167	0.167	0.109
**p value**	0.462	0.486	0.703	0.703	0.886

Supplementary Table S2: Spearman correlation of the percentage of $CD68^+$ cells of all isolated villous cells and different patient characteristics; * significant $p < 0.05$.

VT

Digestion steps	1	2	3
**Time**	15min	20min	1h
**Temperature**	37°C	37°C	37°C
**Trypsin concentration**	1.25mg/ml	0.72mg/ml	-
**Collagenase A concentration**	-	-	1mg/ml
**DNAse I concentration**	0.1mg/ml	0.1mg/ml	0.1mg/ml
**Dilution medium**	HBSS-HEPES	HBSS-HEPES	DMEM

Supplementary Table S3: Digestion steps for villous tissue.

Decidua

Digestion steps	1	2
**Time**	20min	1h
**Temperature**	37°C	37°C
**Trypsin concentration**	0.72mg/ml	-
**Collagenase A concentration**	-	1mg/ml
**DNAse I concentration**	0.02mg/ml	0.02mg/ml
**Dilution medium**	HBSS-HEPES	DMEM

Supplementary Table S4: Digestion steps for Decidua.

	FACS (n=8)	IF/IHC (n=9)
**Gestational age (weeks+days)**	39+3 ± 1+0 (38+0 – 40+6)	37+5 ± 1+5 (35+1 – 40+0)
**Maternal Age (years)**	35 ± 4 (28 - 40)	32 ± 5 (22-39)
**Fetal sex**	♂4 ♀4	♂1 ♀8

Supplementary Table S5: Patient characteristics of FACS and IF results. All data are shown as mean ± SD (range).

Antibody /Dye	Color	Species isotype	Manufacturer	Clone	Used Concentration
**CD45**	FITC	Mouse IgG1, κ	BioLegend (San Diego, USA)	HI30	2.0μg/ml
**CD163**	APC	Mouse IgG1, κ	BioLegend	GHI/61	2.0μg/ml
**CD80**	BV421	Mouse IgG1, κ	BioLegend	2D10	1.6μg/ml
**CD68**	APC-Cy7	Mouse IgG2b, κ	BioLegend	Y1/82A	1.6μg/ml
**CK7**	PE	Rabbit IgG	Abcam (Cambridge, UK)	EPR1619Y	4.0μg/ml
**7AAD**			BD Biosciences (Franklin Lakes, USA)		
**Fixable viability dye eFluor 780**			Thermofisher		

Supplementary Table S6: Antibodies and dyes used for the FACS-staining.

Problem	Possible reason	Solution
**Too less isolated macrophages**	Tissue is too sticky	Wash with enough HBSS-HEPES between the digestion steps.
	Too less Percoll-layers	Use 4 tube with Percoll layer for 150gr. VT-tissue and 2 tubes for whole (ca. 35gr.) Decidua.
**No layer after Percoll centrifugation**	Wrong centrifuge setting	Check the centrifuge settings again, you have to use 2100g at RT without brake.
	Pancoll (1.077 gr./ml) was used instead of Percoll (1.130 gr./ml)	Use Percoll or different centrifuge settings; With the same concentrations and settings like for Percoll, all the cells sink to the bottom of the tube with Pancoll.
**Trophoblast contamination**	Too less Percoll-layers	Use 4 tubes with Percoll layer for 150gr. VT-tissue and 2 tubes for whole (ca. 35gr.) Decidua.
	The red and yellowish top layer from the Percoll tube were not removed	Remove the top red and yellowish layer with a different plastic Pasteur pipette before taking out the macrophages layer.
**Too much red blood cell contamination**	Erythrocyte layer was also aspirated in the pipette	With the help of the plastic Pasteur pipette, the transparent layer is slowly and carefully aspirated. Too fast aspiration leads to the swirling of the erythrocytes. At the end, a thin transparent macrophages layer must be left.
	Still too much blood in the tissue after washing	Scratch the tissue better before washing. Repeat the washing more often with 0.9% NaCl.

Supplementary Table S7: Trouble shooting.

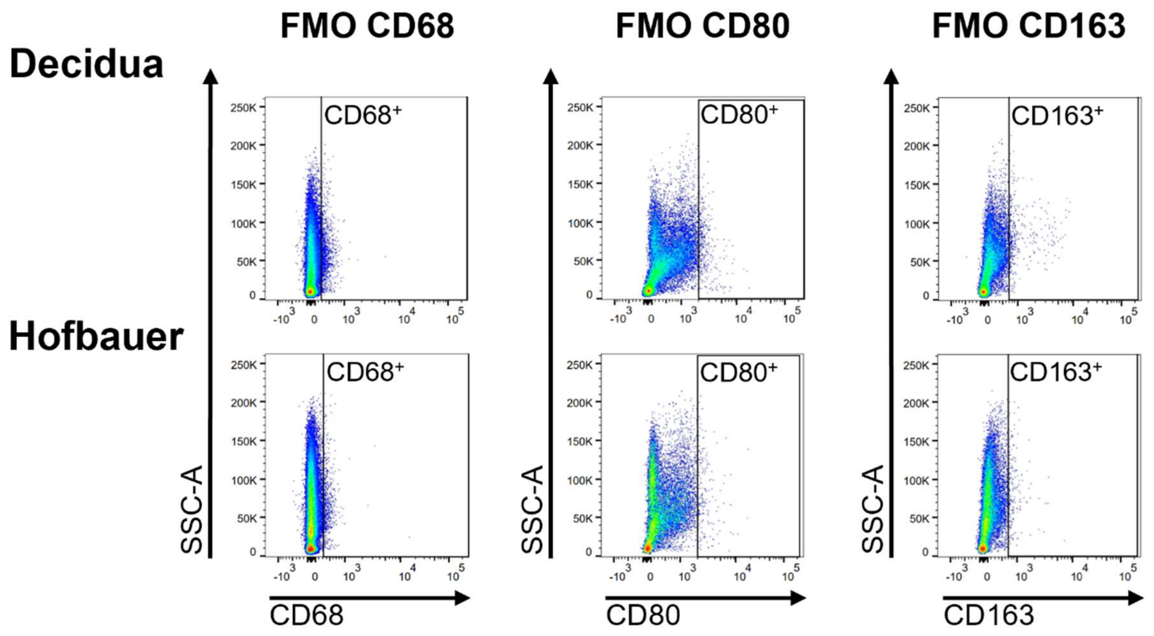

Supplementary Figure S1: FMOs for decidual and Hofbauer macrophages.

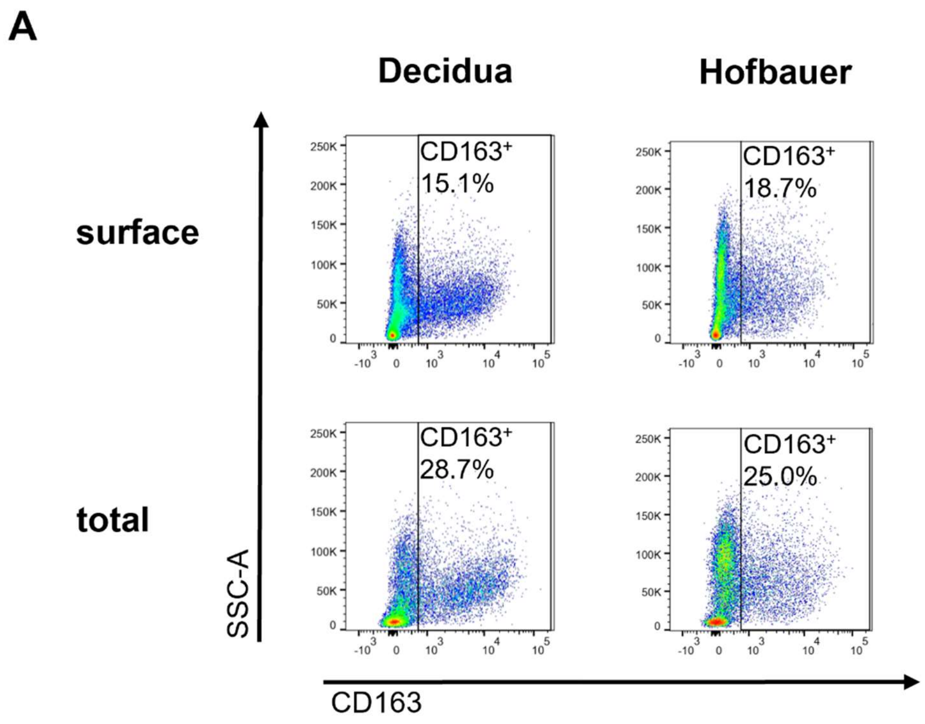

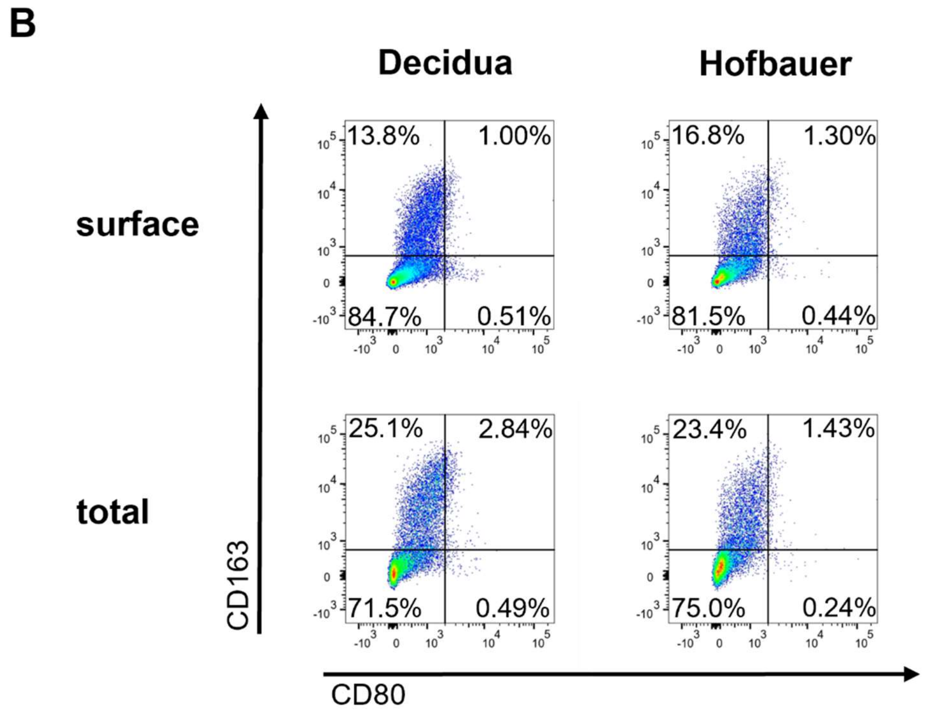

Supplementary Figure S2: Comparison of surface and total staining of CD163: FACS images of (A) $CD163^+$ macrophages and (B) $CD80^+CD163^+$ macrophages.

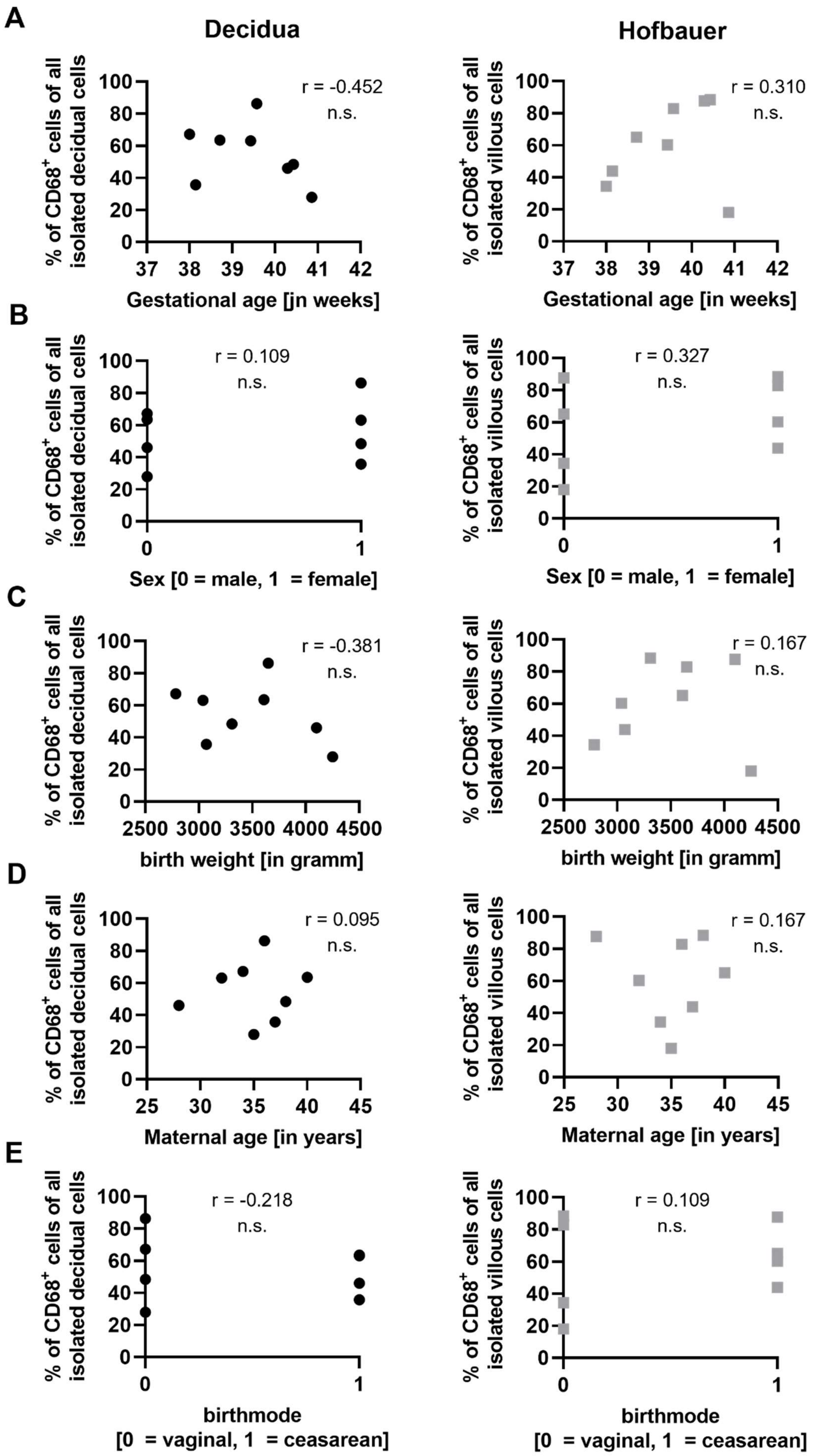
A
Decidua
Hofbauer
% of CD68$^+$ cells of all isolated decidual cells
% of CD68$^+$ cells of all isolated villous cells
r = -0.452
n.s.
r = 0.310
n.s.
Gestational age [jn weeks]
Gestational age [in weeks]
B
r = 0.109
n.s.
r = 0.327
n.s.
Sex [0 = male, 1 = female]
C
r = -0.381
n.s.
r = 0.167
n.s.
birth weight [in gramm]
D
r = 0.095
n.s.
r = 0.167
n.s.
Maternal age [in years]
E
r = -0.218
n.s.
r = 0.109
n.s.
birthmode
[0 = vaginal, 1 = ceasarean]

Supplementary Figure S3: Spearman correlation of the percentage of CD68⁺ of all isolated cells of decidual and villous tissue and (A) the gestational age, (B) fetal sex, (C) birth weight, (D) maternal age and (E) birthmode; * significant $p < 0.05$.

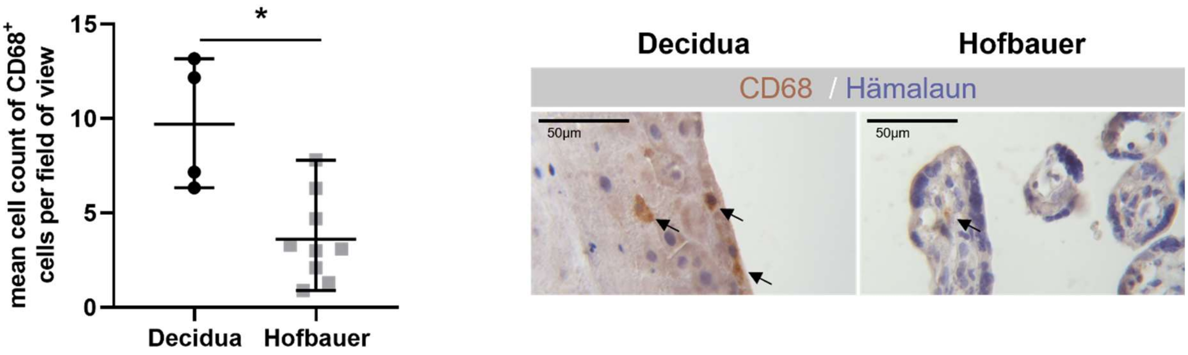

Supplementary Figure S4: Differences in the mean cell count of CD68⁺ cells per field of view in the IHC of CD68; Doplots (as mean ± range) and representative IHC-images; p-values were calculated with Mann-Whitney-U-Test, * significant $p < 0.05$; n=9.
